# A Celestial Assisted INS Initialization Method for Lunar Explorers

**DOI:** 10.3390/s110706991

**Published:** 2011-07-04

**Authors:** Xiaolin Ning, Longhua Wang, Weiren Wu, Jiancheng Fang

**Affiliations:** School of Instrumentation Science & Opto-electronics Engineering, BeiHang University (BUAA), Beijing 100191, China; E-Mails: beihang456wlh@126.com (L.W.); wuwr2002@vip.sina.com (W.W.); fangjiancheng@buaa.edu.cn (J.F.)

**Keywords:** lunar exploration, autonomous initialization, inertial navigation, celestial navigation

## Abstract

The second and third phases of the Chinese Lunar Exploration Program (CLEP) are planning to achieve Moon landing, surface exploration and automated sample return. In these missions, the inertial navigation system (INS) and celestial navigation system (CNS) are two indispensable autonomous navigation systems which can compensate for limitations in the ground based navigation system. The accurate initialization of the INS and the precise calibration of the CNS are needed in order to achieve high navigation accuracy. Neither the INS nor the CNS can solve the above problems using the ground controllers or by themselves on the lunar surface. However, since they are complementary to each other, these problems can be solved by combining them together. A new celestial assisted INS initialization method is presented, in which the initial position and attitude of the explorer as well as the inertial sensors’ biases are estimated by aiding the INS with celestial measurements. Furthermore, the systematic error of the CNS is also corrected by the help of INS measurements. Simulations show that the maximum error in position is 300 m and in attitude 40″, which demonstrates this method is a promising and attractive scheme for explorers on the lunar surface.

## 1. Introduction

The Moon is the only natural satellite of Earth. There is great potential to develop new technologies and to make use of the Moon’s valuable resources. Up to now, the Moon has been visited by the explorers of the Soviet Union (SU), the United States (US), the European Space Agency (ESA), Japan (JP), China (CHN) and India (IN). There are many lunar exploration programs currently happening or being planned.

China’s lunar exploration is a three-phase mission. In phases I and II, China launched its first and second lunar probes, Chang’e-1 and Chang’e-2, which have successfully finished their missions and obtained 3D images of the lunar surface. In the next few years of the phase II, an unmanned lander, which will carry a lunar rover for the exploration of the Moon’s surface, will softly land on the Moon. In phase III, a return vehicle will collect samples of lunar soil and carry them back to the Earth. In these upcoming missions to the Moon, explorers such as Rovers, Landers, Descenders and Ascenders will use INS and CNS for navigation to compensate for the limited capacity of ground tracking networks. The accuracy of INS and CNS mainly depends upon the accuracy with which these systems are initialized or calibrated, so the accurate initialization of the INS and the precise calibration of the CNS are needed.

The initialization of INS is the process of determining some initial values of the system, such as position, attitude, and sensors biases [1,2]. The calibration of CNS is the process of determining and correcting systematic error caused by sensors index error and alignment error [3]. The initialization of the INS and the calibration of the CNS are difficult problems on the lunar surface and cannot be accomplished by each one alone. However, because INS and CNS have complementary characteristics, their initialization and calibration can be accomplished by the combination of them [4].

A celestial assisted INS initialization method for explorers on the lunar surface is presented. An unscented Kalman filter is used for fusing information from various INS and CNS sensors. The initial position, attitude as well as biases of INS sensors are estimated effectively and the systematic error of CNS is corrected at the same time. The feasibility of this new method is validated using a ground test bed. Simulations show that the maximum error in position is 300 m and in attitude 40″. These results verify that this method is a promising and attractive scheme for lunar explorers.

This paper is systematized in five sections. After this introduction, the basic principle of INS and CNS is outlined in Section 2. Then the state model and measurement model of this celestial assisted INS initialization method is described in details in Section 3. Simulations in Section 4 demonstrate the performance and conclusions are drawn in Section 5.

## 2. Inertial Navigation System and Celestial Navigation System

### 2.1. Reference Frames

**Figure 1.**
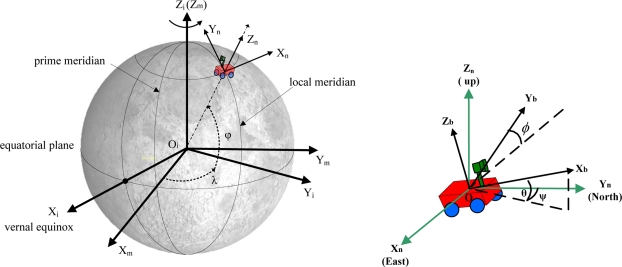
Reference frames.

Reference frames used in this paper are defined as follows:

1. The inertial frame (*O_i_X_i_Y_i_Z_i_*). As shown in Figure 1, the inertial frame has its origin at the center of the Moon. Its z-axis is normal to the equatorial plane, x-axis is in the equatorial plane and points to the vernal equinox, and the y-axis completes a right-handed orthogonal frame.
2. The Moon fixed frame (*O_m_X_m_Y_m_Z_m_*). The Moon fixed frame has its origin at the center of the Moon. Its z-axis is normal to the equatorial plane, x-axis is in the equatorial plane and points to the prime meridian (0° longitude), and the y-axis completes a right-handed orthogonal frame.
3. The navigation frame (*O_n_X_n_Y_n_Z_n_*). The navigation frame is a local vertical frame and has its origin at the location of the explorer. Its x-axis points to the east, the y-axis points to the north, and the z-axis points upward [5].
4. The explorer body frame (*O_b_X_b_Y_b_Z_b_*). The explorer body frame is rigidly attached to the explorer and has its origin at the center of the mass of the explorer. Its x-axis is in the symmetry plane of the body pointing at the direction that the explorer moves along. Its z-axis is perpendicular with the symmetrical plane of the body and points down and the y-axis completes a right-handed orthogonal frame. The rotation around these axes defines the angle of roll(*φ*), pitch(*θ*), and yaw(*ψ*), respectively (Figure 1).

### 2.2. The Inertial Navigation System (INS) and Its Initialization

An INS usually includes a navigation computer and an inertial measurement unit (IMU), which typically consists of three orthogonal accelerometers and three orthogonal gyroscopes. By tracking both the current angular velocity and the current linear acceleration of the explorer measured by IMU, the INS determines the linear acceleration of the explorer in the inertial frame. Thus, if the original velocity and position are known, the inertial velocity of the explorer can be obtained by integration of the inertial acceleration, and integration again yields the inertial position. There are two types of inertial navigation systems: platform inertial navigation system and strap-down inertial navigation system (SINS). In the platform inertial navigation system, IMU is mounted on a mechanical platform, which can isolate explorer’s motion and is held in alignment with the expected navigation frame. The main disadvantages of this system are that the mechanical platform is expensive and its moving parts tend to wear out or jam. In the SINS, IMU is mounted rigidly onto the explorer, and a mathematical platform takes the place of the mechanical platform. This reduces the cost and size, increases the reliability by eliminating the moving parts. In this study, SINS is used for navigation of the lunar explorer. The basic equations of inertial navigation in the navigation frame can be simply expressed as follows [6]:

(1) $$\begin{array}{l} \{\begin{array}{l} \dot{r}\;=\;Dv\\ \dot{v}\;=\;R_{b}^{n}\;f^{b}\;-\;(2w_{\mathrm{im}}^{n}\;+\;w_{\mathrm{mn}}^{n})\;\times \;v\;-\;g\\ {\dot{R}}_{b}^{n}\;=\;-\Omega_{\mathrm{bn}}^{n}R_{b}^{n} \end{array}\\ D\;=\;\left[\begin{array}{ccc} 1/R_{m} & 0 & 0\\ 0 & 1/(R_{m}\;\text{cos}\;L) & 0\\ 0 & 0 & 1 \end{array}\right] \end{array}$$

where *r* = [*L*, *λ*, *h*]*^T^* is the explorer’s position vector, *L*, *λ*, *h* are explorer’s latitude, longitude and altitude in the Moon fixed frame. *v =* [*v_x_*, *v_y_*, *v_z_*]*^T^* is the explorer’s velocity vector. *f^b^* is the output of accelerometers. $w_{\mathrm{im}}^{n}\;=\;{\left[0,\;w_{m}\;\text{cos}\;L,\;w_{m}\;\text{sin}\;L\right]}^{T}$ is the rotation rate vector of the Moon fixed frame with respect to the inertial frame. *w_m_* is the magnitude of the rotation rate of the Moon and has the value 2.66 × 10^−6^ rad/s. $w_{\mathrm{mn}}^{n}\;=\;{\left[-v_{y}/R_{m},\;v_{x}/R_{m},\;v_{x}\;\text{tan}(L)/R_{m}\right]}^{T}$ is the rotation rate vector of the navigation frame with respect to the Moon fixed frame. *R_m_* is the radius of the Moon and has the value 1,738 km. *g* = [0,0,1.618*m*/*s*^2^]*^T^* is the gravity vector. $\Omega_{\mathrm{bn}}^{n}$ is a skew symmetric matrix which can be described by the gyro outputs $w_{\mathrm{ib}}^{b}$ and $w_{\mathrm{in}}^{n}$ The superscript *n* and *b* denote the navigation frame and the explorer body frame, respectively. $R_{b}^{n}$ is the transformation matrix from the explorer body frame to the navigation frame, which is also the attitude matrix and can be defined as

(2) $$R_{b}^{n}\;=\;\left[\begin{array}{ccc} \text{cos}\;\varphi \;\text{cos}\;\Psi \;-\;\text{sin}\;\varphi \;\text{sin}\;\Psi \;\text{sin}\;\theta \; & -\;\text{sin}\;\Psi \;\text{cos}\;\theta & \text{cos}\;\Psi \;\text{sin}\;\varphi \;+\;\text{sin}\;\Psi \;\text{sin}\;\theta \;\text{cos}\;\theta \\ \text{cos}\;\varphi \;\text{sin}\;\Psi \;+\;\text{sin}\;\varphi \;\text{cos}\;\Psi \;\text{sin}\;\theta & \text{cos}\;\Psi \;\text{cos}\;\theta & \text{sin}\;\Psi \;\text{sin}\;\varphi \;-\;\text{cos}\;\Psi \;\text{sin}\;\theta \;\text{cos}\;\varphi \\ -\;\text{cos}\;\theta \;\text{sin}\;\varphi & \text{sin}\;\theta & \text{cos}\;\theta \;\text{cos}\;\varphi \end{array}\right]$$

From the principle of INS we can see that an initialization is needed before INS can properly work. INS initialization is the process of determining initial values for position, velocity, and attitude in the navigation frame, and in some cases, inertial sensor errors are also estimated. INS attitude initialization is called alignment, which is the process of determining the initial values of the coordinate transformation from the body frame to the navigation frame in SINS [1].

As a relatively mature technology, initial alignment of INS on the Earth has been widely studied in the literature. The main research directions include: INS error models [7–9], filter methods [10,11], observability analysis [12,13], and *etc.* The basic principle that most methods and techniques are based on is as follows. INS is initially provided with its position from a human operator or GPS, *etc.* Its initial velocity can be set to zero if it starts from rest. The alignment can be accomplished by sensed gravity and Earth’s rotation vectors [14,15]. However, on the lunar surface, the accuracy of position provided by the ground tracking is only about 1,000 m. Though the gravity information is available, the lunar rotation rate is so small that its accuracy of measurement is not high enough for executing alignment. Thus, there must be other means to execute the INS initialization. It also can be seen that small measurement errors introduced by accelerometers and gyroscopes are integrated into large errors in velocity and position. These errors are cumulative and increase with time. The errors existing in inertial sensors include zero-mean random errors and systematic errors. Systematic errors, such as scale factor and bias variations can be modeled and calibrated. In this study, only biases are calibrated in the initialization.

### 2.3. Celestial Navigation System (CNS) and Its Calibration

**Figure 2.**
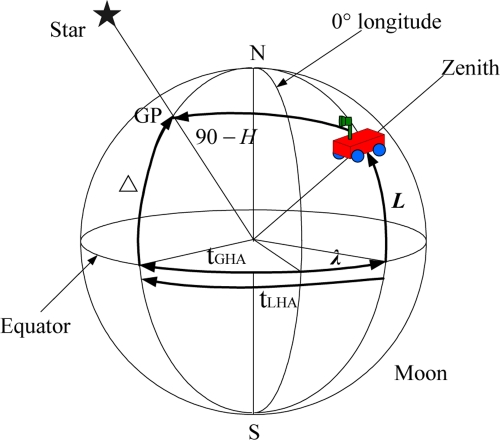
Parameters in the Moon fixed frame.

Stars always move in the regular way, their positions can be known exactly at a specific time. Celestial navigation is a kind of technology of finding one’s position through astronomical observations. CNS is usually comprised of a star sensor (or sun sensor) and an inclinometer. The star sensor is used to measure the direction of the star and the inclinometer is used to measure local vertical direction. Thus the star altitude, which is the angle between the horizon and the line of sight to the star, can be subtended. The star altitude is a function of the explorer’s position and the geographic position (GP) of the star, which is expressed in the Moon fixed frame as follows:

(3) $$\{\begin{array}{c} \text{sin}\;L\;\cdot \;\text{sin}\;\Delta \;+\;\text{cos}\;L\;\cdot \;\text{cos}\;\Delta \;\cdot \;\text{cos}\;t_{\mathrm{LHA}}\;=\;\text{sin}\;H\\ t_{\mathrm{LHA}}\;=\;t_{\mathrm{GHA}}\;+\;\lambda \end{array}$$

where *H* is the star altitude. Δ*t_LHA_* are the declination and local hour angle of the star. *t_LHA_* is the sum of *λ* and *t_GHA_*. *t_GHA_* is the angular distance to the meridian of the GP measured westward from the 0° longitude. The GP of a star can be determined by its Δ and *t_LHA_*, which can be obtained from the Astronomical Almanac together with the precise measurement time. The geometric meanings of these parameters are shown in Figure 2.

When there are enough measurements, the explorer’s position can be determined by the intercept method or the filter method [16]. It can be seen from above principle that the navigation accuracy of CNS depends mainly on the accuracy of star altitude measurements. In fact, there are many errors existing in star altitude measurements, such as sensors (for both the inclinometer and the star sensor) index error, signal noise, and alignment error. These errors can significantly degrade navigation accuracy. Previous study shows that 5.93″ is the maximum allowable lumped error that will ensure accuracy of coordinate results to within 50 m [17]. These errors can be broadly divided into two types: systematic error and random error. Systematic error is usually constant and can be modeled by mathematic functions. Random error is the unpredictable error and cannot be modeled. In this study, the systematic error in CNS measurement is estimated and corrected by the help of INS measurements using following method.

## 3. Celestial Assisted INS Initialization Method

The traditional INS initialization method on the earth based on Kalman filter usually uses the INS error model as the state model [18,19]. The state variables of this model include two horizontal position errors, two horizontal velocity errors, three attitude error angles, three gyro biases and three accelerometer biases. Measurements include velocity error, Earth rotation rate, *etc.* The observability of this method is low and many state variables are not observable. Because CNS can provide refined position and attitude information, CNS aided INS initialization not only improves the observability, but also improves the initialization accuracy. To make system models simple, two horizontal positions, three attitude angles, three gyro biases, three accelerometer biases are used as state variables. In addition to these eleven variables, the systematic error in the star altitude measurement is also added to the state variables.

### 3.1. State Model

Because the lunar explorer is stationary, the state model in the Moon fixed frame is defined as:

(4) $$\begin{array}{c} \dot{X}\;=\;0\\ X\;=\;{\left[L,\;\lambda ,\;\theta ,\;\varphi ,\;\psi ,\;b_{x},\;b_{y},\;b_{z},\;a_{x},\;a_{y},\;a_{z},\;\alpha \right]}^{T} \end{array}$$

where *L*, *λ* are lunar explorer’s position (latitude and longitude). *θ*, *φ*, *ψ* are attitude Eula angles. *b_x_*,*b_y_*,*b_z_* are gyros errors; *a_x_*,*a_y_*,*a_z_* are accelerometers errors; both errors are simply modeled as a constant [20]. *α* is the systematic error in the star altitude measurement, which also can be modeled as a constant.

### 3.2. Measurement Model

To make all state variables observable, the star altitude, star orientation and outputs of IMU are chosen as the measurement variables.

1. Star altitude. From Equation (3), the measurement equation of star height *H* is obtained as:
  (5) $$\begin{array}{c} H\;=\;\text{arcsin}(\text{sin}\phi \;\cdot \;\text{sin}\;\Delta \;+\;\text{cos}\;\varphi \;\cdot \;\text{cos}\;\Delta \;\cdot \;\text{cos}\;t_{\mathrm{LHA}})\;+\;\alpha \;+\;v_{H}\\ t_{\mathrm{LHA}}\;=\;t_{\mathrm{GHA}}\;+\;\lambda \\ \phi \;=\;\phi_{e}\;+\;\Delta \phi \\ \lambda \;=\;\lambda_{e}\;+\;\Delta \lambda \end{array}$$
  where *λ_e_*, *ϕ_e_* are the pre-estimated longitude and latitude of lunar probe respectively, which is provided by ground tracking network. The error between *λ_e_*, *ϕ_e_* and the true value is about 1,000 m. *α* is the systematic error in the star altitude measurement as mentioned above and *v_H_* is measurement noise.
2. Star direction vector. The observation information measured by star sensor also provides an indication of the lunar explorer’s attitude information. Given the 2-D star centroid from the threshold star image, a 3-D star-direction unit vector *s_b_* = [*x_b_*,*y_b_*,*z_b_*]*^T^* in the explorer body frame can be computed [21]. At the same time, the vector direction of star in the Moon fixed frame can be obtained from the Astronomical Almanac, given as follows: *s_m_* = [*x_m_*,*y_m_*,*z_m_*]*^T^* = [cosΔcos *t_LHA_*, cosΔsin *t_LHA_*, sinΔ]*^T^* The relation between *s_b_* and *s_m_* is:
  (6) $$s_{m}\;=\;R_{n}^{m}\;\cdot \;R_{b}^{n}\;\cdot \;s_{b}$$
  where $R_{n}^{m}$ is the transformation matrix from the navigation frame to the Moon fixed frame:
  (7) $$R_{n}^{m}\;=\;\left[\begin{array}{ccc} -\text{sin}\;\lambda & -\text{sin}\;L\;\text{cos}\;\lambda & \text{cos}\;L\;\text{cos}\;\lambda \\ \text{cos}\;\lambda & -\text{sin}\;L\;\text{sin}\;\lambda & \text{cos}\;L\;\text{sin}\;\lambda \\ 0 & \text{cos}\;L & \text{sin}\;L \end{array}\right]$$
3. Output of accelerometers:
  (8) $$\begin{array}{c} f^{b}\;=\;R_{n}^{b}\;\cdot \;g_{t}\;+\;g_{\Delta }\\ g_{t}\;=\;{\left[0,0,1.618\right]}^{T}\\ g_{\Delta }\;=\;{\left[a_{x},\;a_{y},\;a_{z}\right]}^{T} \end{array}$$
  where *f^b^* is the output of accelerometers, *g_t_* is gravity vector, *g*_Δ_ is the vector of accelerometers biases.
4. Output of gyros:
  (9) $$\begin{array}{c} w^{b}\;=\;R_{n}^{b}\;\cdot \;R_{m}^{n}\;\cdot \;w_{t}\;+\;w_{\Delta }\\ w_{t}\;=\;{\left[0,\;0,\;2.6617e-6\right]}^{T}\\ w_{\Delta }\;=\;{\left[b_{x},\;b_{y},\;b_{z}\right]}^{T} \end{array}$$
  where *w^b^* is the output of gyros, *w_t_* is the lunar rotation rate, *w*_Δ_ is the vector of gyros biases. Using Equations (5–9), we can obtain the following measurement model of this celestial assisted INS initialization method:
  (10) $$Z\;=\;[H,\;s_{b},\;f^{b},\;w^{b}]$$

### 3.3. UKF Method

**Figure 3.**
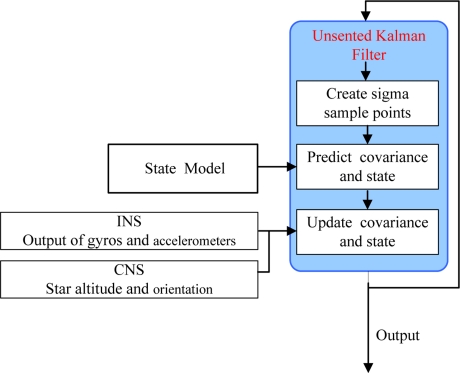
Celestial assisted INS initialization algorithm.

The Kalman filter (KF), which is optimal for application to linear and Gaussian systems, is often used in the INS initialization [22]. However, in this celestial assisted INS initialization system, the measurement model is nonlinear. Since UKF has lower estimation errors than the Extended Kalman filter (EKF) for nonlinear systems and it also avoids the derivation of Jacobian matrices, UKF is used in this study [23,24]. The block diagram of the celestial assisted INS initialization method based on UKF is shown in Figure 3.

## 4. Results and Discussion

**Figure 4.**
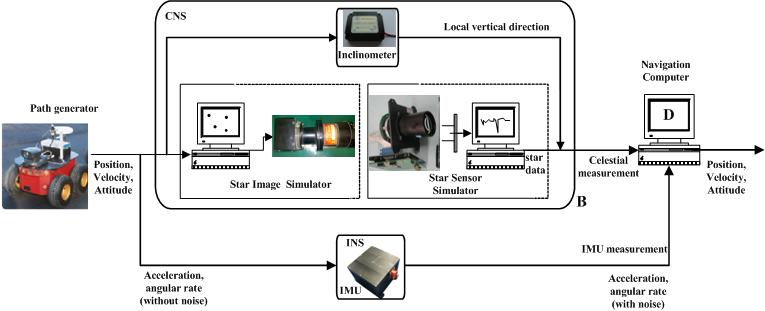
Lunar explorer INS/CNS simulation system.

This section presents simulations of this celestial assisted INS initialization method. All simulation data comes from the lunar explorer INS/CNS simulation system shown in Figure 4. This system is composed of a lunar explorer simulator, a real-world model characterizing both navigation environment and on-board sensors and a navigation computer. A pioneer-3A robot is used as the lunar explorer simulator to evaluate navigation performance. The INS is composed of an IMU, which is composed of three optical fiber gyroscopes and three quartz pendulum accelerometers. The IMU is rigidly mounted parallel to the body axes of the explorer. The bias of each gyroscope is 0.05°/h and the bias of each accelerometer is 10 μg. The update rates of both sensors are 100 Hz. The CNS consists of a star image simulator, a star sensor and an inclinometer. The star image simulator is used to produce the simulation star image according to the explorer’s position, attitude, the Astronomical Almanac and astronomy software, *etc.* The accuracy of star sensor is 3″ (1σ) and its update rate is 5 Hz. The inclinometer used is a NS–45/P2 dual-axis inclinometer. The precision of the inclinometer is 0.03° and its update rate is 20 Hz.

**Figure 5.**
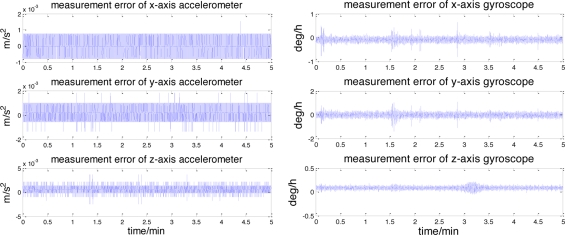
Accelerometer and gyro errors.

**Figure 6.**
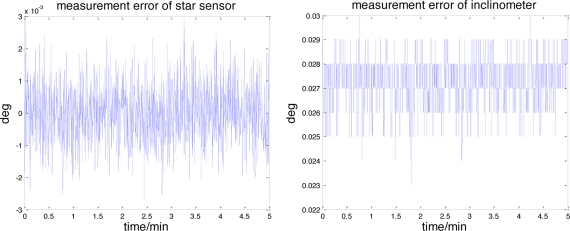
The measurement errors of the star sensor and the inclinometer.

In simulations, measurement errors are separated from the real data and used to create simulation measurements on the lunar surface. The error characteristics of the accelerometers and gyroscopes are shown in Figure 5. It can be seen that each accelerometer measurement error consists of a constant bias and the random noise, which can also be observed in the gyroscopes. The raw measurement errors of the star sensor and the inclinometer are depicted in Figure 6.

Similar to Figure 5, the dominant components of these errors also consists of the constant bias and the random noise. The landsite of USA Surveyor III (2°56′N, 336°40′E) is chosen as the initial position. The lunar rover stays still, its initial yaw, roll and pitch angles are 20°, 0° and 0°. The entire simulation time is 5 min.

**Figure 7.**
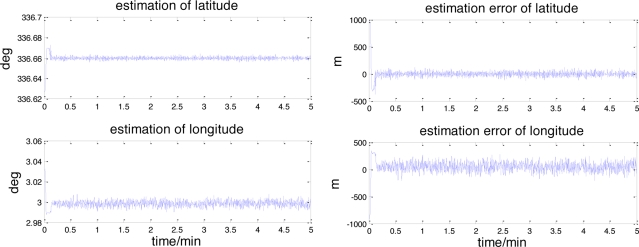
The position estimation and its error.

**Figure 8.**
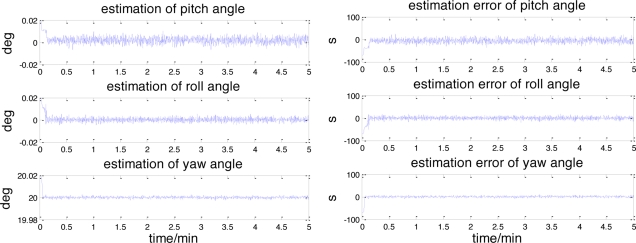
The attitude estimation and its error.

Figure 7 and Figure 8 shows the results of this celestial assisted INS initialization method. As indicated in these figures, the UKF filter converges rapidly within 10 seconds from the start of the run. After the filter convergence period, the estimated values of latitude and longitude converge to 336.66° and 2.9984° quickly, which are very close to the true value 336.6585° and 3.0023°. The root mean square (RMS) estimation errors of latitude and longitude are 39.9239 m and 90.4737 m. The maximum estimation errors of latitude and longitude are 131.8504 m and 276.1276 m.

The estimated attitude also converges to the real attitude quickly. The root mean square (RMS) attitude errors are 11.3343″, 5.9231″ and 2.8087″ respectively in yaw, roll and pitch angle. The maximum estimation errors of these angles are 36.3830″, 19.7239″ and 10.2493″. From these results, it can be concluded that this celestial assisted INS initialization method can enhance the position and attitude estimation accuracy. The maximum error in position is 300 m and in attitude 40″, which is much better than that sent by ground stations.

**Figure 9.**
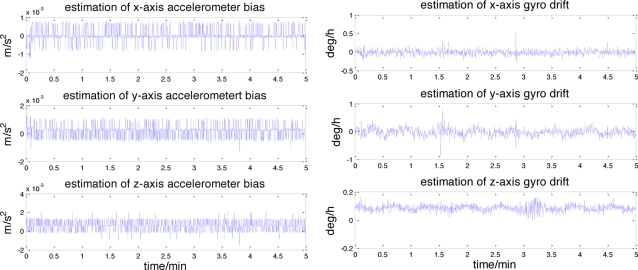
The estimation of accelerometer and gyroscope errors

**Figure 10.**
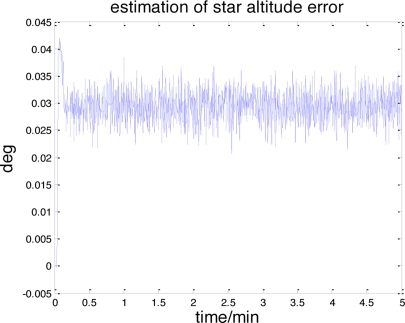
The estimation of star altitude error.

Figure 9 illustrates the accelerometer and gyroscope errors estimated by this celestial assisted INS initialization method. These results are coherent with the true measurement errors, as evidenced in Figure 5. The systematic error estimation result of star altitude in the CNS is presented in Figure 10. Compared with Figure 6, we can see that the estimation is consistent with the measurement error caused by the star sensor and the inclinometer. From these figures, it is clear that the celestial assisted INS initialization method developed in this paper is able to accurately estimate the accelerometer and gyroscope errors. Furthermore, it is also can estimate the systematic error in the CNS effectively.

## 5. Conclusions

In this study, a new celestial assisted INS initialization method for lunar explorers, which could solve the INS initialization and CNS calibration problems at the same time, is presented. To make the state model simple and observable, position, attitude, and main error resources are used as state variables. All original information provided by the sensors of the INS and the CNS is used as measurements. An unscented Kalman filter is used to deal with these measurements and estimate the states. The method is tested using a ground simulation system. The estimation error of initial position is within 300 m and the estimation error of initial attitude is within 40″. Both the inertial sensors’ biases in the INS and the systematic error in the CNS are estimated effectively. Results allow the conclusion that this celestial assisted INS initialization method is a promising method for the high accuracy initialization of a lunar explorer. It should be noted that the proposed approach is general and may be used on any kind of lunar explorers on the lunar surface, such as lunar rovers, landers and ascenders, with an equivalent set of sensors. Future directions of research include applications (extensions) of this method to cases where the INS and CNS are fused with other navigation sensors (e.g., vision sensors, beacons *etc.*)
